# Multi‐ancestry whole genome sequencing analysis of 36,361 individuals from the Alzheimer’s Disease Sequencing Project (ADSP)

**DOI:** 10.1002/alz.092063

**Published:** 2025-01-03

**Authors:** Brian W. Kunkle, John J. Farrell, Bilcag Akgun, Dongyu Wang, Congcong Zhu, Kara L. Hamilton‐Nelson, Sven‐Thorsten Dietrich, Xiaoling Zhang, Andy Rampersaud, Jiemin Yang, Adam C. Naj, Eden R. Martin, Anita L. DeStefano, Seung Hoan Choi, the Alzheimer’s Disease Sequencing Project (ADSP)

**Affiliations:** ^1^ John P. Hussman Institute for Human Genomics, University of Miami Miller School of Medicine, Miami, FL USA; ^2^ Biomedical Genetics, Boston University Medical School, Boston, MA USA; ^3^ Boston University School of Public Health, Boston, MA USA; ^4^ Department of Medicine (Biomedical Genetics), Boston University Chobanian & Avedisian School of Medicine, Boston, MA USA; ^5^ Research Computing Services, Boston University, Boston, MA USA; ^6^ Penn Neurodegeneration Genomics Center, University of Pennsylvania Perelman School of Medicine, Philadelphia, PA USA

## Abstract

**Background:**

The Alzheimer’s Disease Sequencing Project (ADSP) aims to identify genetic variation contributing to the development or protection of Alzheimer’s disease (AD) in diverse ancestral populations. The latest ADSP whole genome sequencing (WGS) data release includes over 36,000 individuals from 37 datasets (NIAGADS NG00067.v11 ADSP R4). Quality control (QC) of these data has been performed and association analyses are underway.

**Method:**

We performed QC filtering based on metrics including GATK VQSR, genotype missingness rate (<20%), comtamination rate, singleton rate, heterozygous/homozygous ratio, ABHet ratio ([0.3, 0.7]), Ti/Tv ratio, and a differential allele frequency between PCR‐free and PCR‐amplification library protocol. After filtering, two genome‐wide association analytical pipelines are being implemented: 1) a population‐specific analysis using a Gaussian mixture model genetic clustering approach to identify genetic similarity clusters for defining populations, and 2) a pooled analysis of all populations accounting for population structure and empirical relationships using GENESIS KING, PC‐Air and PC‐Relate methods. Using SAIGE, planned analyses include single variant logistic mixed model analysis and set‐based association tests (Burden, SKAT, SKAT‐O) for coding and noncoding rare variant groups.

**Result:**

After QC, we retained over 293 million single nucleotide variants (SNVs), 15 million small deletions, 6 million small insertions, and over 11,000 AD and related dementia (ADRD) cases, for downstream association analysis. With high‐quality non‐correlated variants (n = xx), we defined 8 genetically similar population clusters for population‐specific analyses comprising ∼800 Amish, ∼12,200 European, ∼5,400 African/African American, ∼5,700 Hispanic/Amerindian/African, ∼2,500 Hispanic/Amerindian, ∼1,200 Ashkenazi Jewish, ∼2,600 Indian, and ∼200 other individuals. In the principal component analysis, the Ashkenazi and Amish population revealed distinct clusters separate from the European reference population.

**Conclusion:**

A major goal of the ADSP is to fully reveal the genetic architecture of AD/ADRD across diverse ancestral populations with the hope that new discoveries and the therapeutic or prevention strategies they enable will benefit all ancestral groups. Complementary pooled‐population and population‐specific analyses of the ADSP’s latest WGS data release are underway and new insights from these investigations are anticipated.

